# The measurement of consciousness: assuming completeness of first-person report significantly restricts scope and reliability of theory-discrimination

**DOI:** 10.3389/fpsyg.2015.00025

**Published:** 2015-02-09

**Authors:** Nicholas M. Rosseinsky

**Affiliations:** Department of Neuroscience, Center for Dialog in ScienceLondon, UK

**Keywords:** measurement, correlates, consciousness, causal closure, first-person report

## Introduction

The target of this comment is a recent and welcome paper (Gamez, 2014) that addresses foundational issues for neural-correlates-of-consciousness empiricism. This comment discusses whether Gamez' assumptions actually allow the suggested framework to support the kind of theory-discrimination necessary for advancing the field. Specifically, present discussions solely concern the theory-discrimination consequences of assumption A4, which states that all detailed features of conscious experience are first-person-reportable (at least, in principle). The present claim is that A4 limits subsequent theory-discrimination such that Gamez' approach is incapable of addressing certain centrally-significant controversies in the field. This claim is not meant to demean the value of Gamez' contribution, but rather to draw attention to still-unresolved issues. The primary challenges to A4 justified and discussed here are explicit cases in which certain details of conscious experience are *not* fully reportable (although these details are of course experienced by the subject).

## Orienting example of a theory-discrimination problem in consciousness research

**Figure 1:**
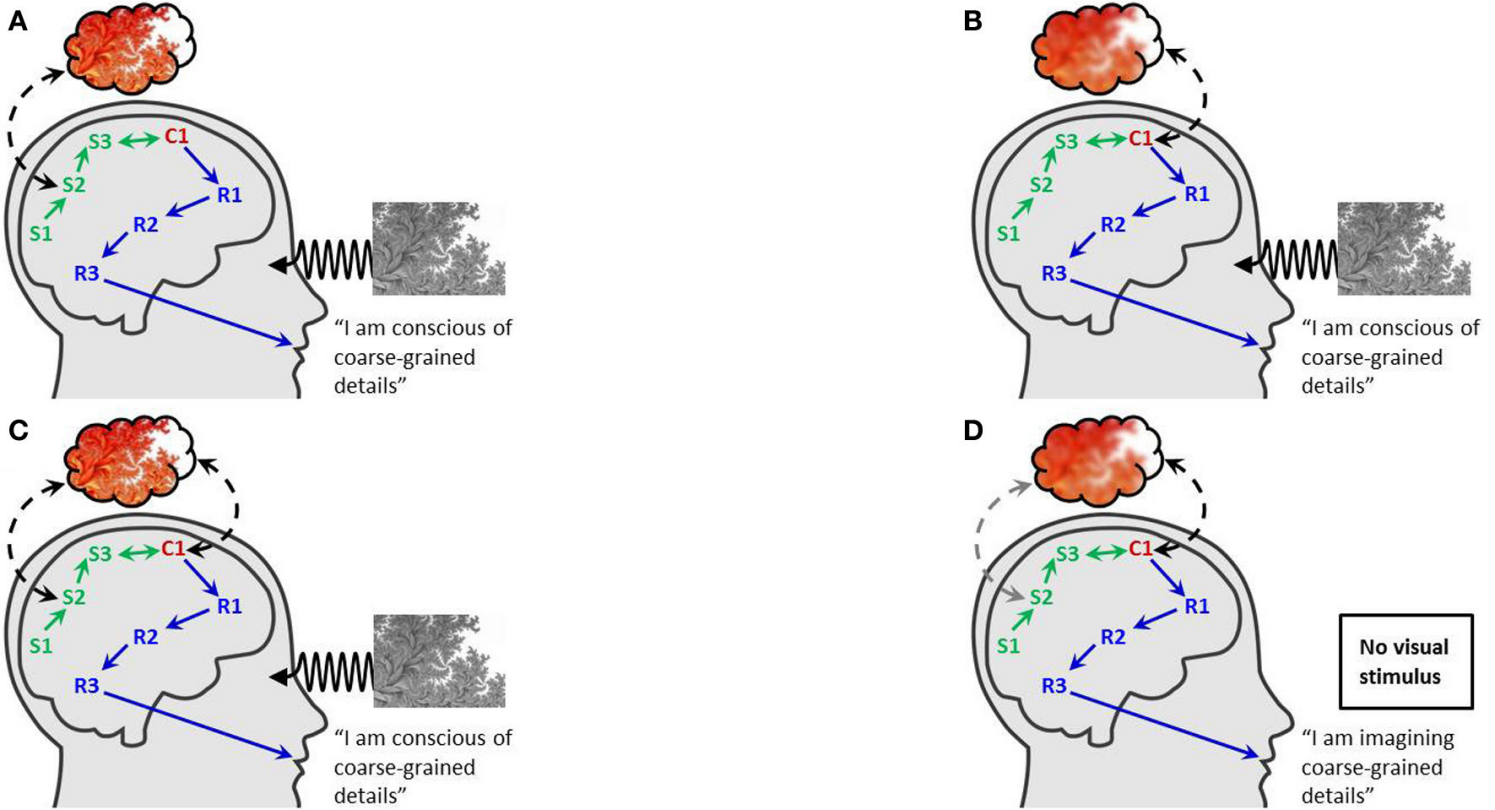
**Assuming completeness of first-person report can lead to unreliable neural-correlates conclusions**. Pictorial conventions and notation follow Figure 4 in Gamez (2014). **(A)** “Theory-I”: conscious experience is directly associated with a relatively early area S2 that encodes the visual scene at a fine-grained resolution; report is based on coarse-grained resolution encoded in C1, and does not fully reflect experience. **(B)** “Theory-II”: conscious experience is directly associated with the later area C1 that encodes at a coarse-grained resolution; report is based on coarse-grained resolution encoded in C1. If completeness of first-person report is assumed, a neural-correlates approach collecting first-person and brain-dynamical data (*identical* in **A,B**) leads to false certainty that theory-II is correct. **(C)** “Theory-III”: conscious experience is associated with both S2 and C1; report is based on C1. For example, S2 might provide fine perceptual detail to conscious experience, while C1 provides context, contours *etc*. **(D)** Imagination of experience, assuming theory-III is correct: if imagination evokes activity in C1 but not S2, and C1 provides only coarse-grained detail, then the imagined experience is not the same as that in the stimulus-driven setting **(C)** (absence of S2-participation in conscious experience schematically indicated by graying of associative arrow). If completeness of first-person report is assumed, a neural-correlates approach contrasting stimulus-driven and imagined experiences **(C,D)** will incorrectly eliminate theory-III in favor of theory-II **(B)**.

Consider two alternative theories for spatiotemporal structures (Gamez, p. 2) directly associated (Gamez, p. 4) with conscious experience (Figures 1A,B). According to theory-I, activity in a relatively early area, S2 say, is directly associated, whereas according to theory-II, the direct associate is activity in a later area C1 that e-causally (Gamez, p. 6) receives information (Shannon, 1948) from S2. By construction of theory-I and theory-II, area S2 dynamically encodes some information concerning the external environment that cannot be decoded from C1-dynamics. Put differently, the e-causal transfer of dynamically-encoded information from S2 to C1 e.g., via S3, loses some details in granularity of representation. (In Figure 1, granularity is illustratively depictedas degree-of-spatial-resolution, although in principle it could relate to any aspect of conscious experience potentially associated with multiple hierarchical representations in brain-dynamical encoding). Crucially, both theory-I and theory-II suppose (by construction) that first-person report is limited to information encoded in C1-activity (because report-governing area R3 is presumed e-causally connected to S2-encoded information only via C1), so that first-person report definitively cannot reflect the granularity available e.g., from detailed third-person decoding of S2-activity using complete knowledge of both S2-dynamics and neural codes. Under Gamez' causality assumptions, neural-correlates-of-consciousness approaches cannot discriminate between theory-I and theory-II in order to establish whether it is S2 or C1 that is directly associated with consciousness, because first-person report and neural dynamics are identical under the two theories (Figures 1A,B).

## Implications for Gamez' framework

The type of theory-discrimination inherent in the theory-I/theory-II example is excluded by Gamez' assumption A4: theory-I contains aspects of conscious experience not available even in-principle for first-person report, and therefore lies outside the framework of Gamez (2014). Thus, the inability of first-person-report-based methodologies to perform theory-I/theory-II discrimination does not invalidate Gamez' claims that his framework provides for consistent empiricism. But this inability does draw attention to limitations not fully emphasized by Gamez, and leads naturally to two questions. First, are theory-discriminations of the theory-I/theory-II kind relevant to advancing the field? (If not, then observations here are only of minor interest, although the relevance of the theory-I/theory-II contrast to *past* controversies in the field follows from direct correspondence with the seminal considerations of Crick and Koch, 1995). Second, if making assumption A4 *does* establish a significant limitation on Gamez' framework, are there alternative assumptions providing a logically-consistent basis for theory-I/theory-II style discriminations whilst preserving rigor and theory-neutrality (Gamez, 2014)?

## Relevance of example-evoked issues to the field

Concerning the first question, one might object that S2-associated theories are implausible, for example because they are not consistent with empirical data. The primary weakness of such objections is that they typically appeal to consistency with first-person report that is precisely at issue in the theory-I/theory-II contrast. For example, one might claim that first-person-reported properties of conscious experience are not consistent with known dynamical-encoding regularities pertaining to S2-activity. But this objection presumes that first-person-report is accurate in full detail, as is precisely excluded by theory-I. Setting aside empirically-based objections, it is straightforward to construct further theory-contrasts that create the same challenges as the example, and are closer to certain central controversies in the field. For example, consider discrimination between theory-II and another theory-III (Figure 1C), that proposes conscious experience is associated with *both* S2 and C1. (For example, C1-activity might be associated with large scale features such as shapes and contours, and S2-activity might provide a supplementary level of vivid detail). Controversies of the theory-II/theory-III kind are certainly longstanding, lively and ongoing (Sperling, 1960; Block, 2007; Cohen and Dennett, 2011; Navajas et al., 2014), thus establishing the relevance of example-evoked issues to the field. Case-by-case demonstration that every empirical method mentioned by Gamez fails discriminatorily is beyond the present article's scope, but Figure 1 illustrates problems for Gamez' closest suggestion for S2/C1-resolution, namely a contrast between stimulus-evoked (Figure 1C) and imagined (Figure 1D) experiences (Gamez, p. 10). Finally, theory-II/theory-III debates go beyond the constraints acknowledged in Gamez (2014) (e.g., the exclusion of micro-consciousness style theories Zeki and Bartels, 1999).

## What assumptions extend the reach of theory-discrimination whilst preserving rigor?

Turning to the second question, note that theory-I/theory-II *discrimination* problems cannot be avoided by adopting identity-theory (Kim, 1998). For example, even assuming conscious experience is in some sense metaphysically identical to neural activity, there is insufficient data (Figures 1A,B) to resolve an S2-identical theory-I from a C1-identical theory-II.

The present article's scope does not admit full exploration of frameworks that can both support theory-discriminations of the kind highlighted here and preserve Gamez' refreshing level of care. To set the stage for future work in this direction, it is perhaps helpful to contrast approaches of Gamez (2014) and Chalmers (1996). Chalmers (Ch. 5) raises problems related to phenomenal judgment, and appeals to a pre-experimental bridging principle based on functionalism (Ch. 6) to exclude S2-style possibilities. As well as compromising Gamez' theory-neutrality by virtue of the functionalist appeal, Chalmers' phenomenal-judgment explanation can be directly criticized for its reliance on non-physical cognition, because this seems to contradict the general tenor of a causal-closure-of-the-physical setting. Gamez' A4 instead excludes S2-style possibilities directly, but limitations on theory-discrimination then return us to Chalmers' original phenomenal-judgment-associated concern: especially under causal closure, what level of objective, scientific, confidence can be given to subjectively-powerful intuitions that brain-based report *does* accurately reflect details of conscious experience?

### Conflict of interest statement

The author declares that the research was conducted in the absence of any commercial or financial relationships that could be construed as a potential conflict of interest.

## References

[B1] Block N. (2007). Consciousness, accessibility, and the mesh between psychology and neuroscience. Behav. Brain Sci. 30, 481–499. 10.1017/S0140525X07002786 18366828

[B2] Chalmers D. J. (1996). The Conscious Mind: In Search of a Fundamental Theory. New York, NY: Oxford University Press.

[B4] Cohen M. A. Dennett D. C. (2011). Consciousness cannot be separated from function. Trends Cogn. Sci. 15, 358–364. 10.1016/j.tics.2011.06.008 21807333

[B5] Crick F. Koch C. (1995). Are we aware of neural activity in primary visual cortex? Nature 375, 121–123. 10.1038/375121a0 7753166

[B6] Gamez D. (2014). The measurement of consciousness: a framework for the scientific study of consciousness. Front. Psychol. 5:714. 10.3389/fpsyg.2014.00714 25071677 PMC4091309

[B7] Kim J. (1998). Mind in a Physical World: An Essay on the Mind-Body Problem and Mental Causation. Cambridge, MA: MIT Press.

[B8] Navajas J. Rey H. G. Quian Quiroga R. (2014). Perceptual and contextual awareness: methodological considerations in the search for the neural correlates of consciousness. Front. Psychol. 5:959. 10.3389/fpsyg.2014.00959 25221537 PMC4148639

[B9] Shannon C. E. (1948). A mathematical theory of communication. Bell Syst. Tech. J. 27, 379–423, 623–656 10.1002/j.1538-7305.1948.tb01338.x

[B10] Sperling G. (1960). The information available in brief visual presentations. Psychol. Monogr. Gen. Appl. 74, 1–29.

[B11] Zeki S. Bartels A. (1999). Towards a theory of visual consciousness. Conscious. Cogn. 8, 225–259. 10.1006/ccog.1999.0390 10448004

